# Antipsychotic Maintenance Treatment: Time to Rethink?

**DOI:** 10.1371/journal.pmed.1001861

**Published:** 2015-08-04

**Authors:** Joanna Moncrieff

**Affiliations:** Division of Psychiatry, University College London, London, United Kingdom

## Abstract

Joanna Moncrieff looks at the lack of long-term evidence for antipsychotic medication and considers what is needed to ensure we have the knowledge to maximize benefits and minimize harms.

**Summary Points**

- Existing studies of long-term antipsychotic treatment for people with schizophrenia and related conditions are too short and have ignored the impact of discontinuation-related adverse effects.
- Recent evidence confirms that antipsychotics have a range of serious adverse effects, including reduction of brain volume.
- The first really long-term follow-up of a randomised trial found that patients with first-episode psychosis who had been allocated to a gradual antipsychotic reduction and discontinuation programme had better functioning at seven-year follow-up than those allocated to maintenance treatment, with no increase in relapse.
- Further studies with long-term follow-up and a range of outcomes should be conducted on alternatives to antipsychotic maintenance treatment for people with recurrent psychotic conditions.

Schizophrenia and psychotic disorders are estimated to affect 1% of the population and are one of the highest causes of global disability [1]. They place a considerable burden on individuals, families, and society, with costs amounting to US$62.7 billion in the United States in 2002, for example [2]. The highest costs are related to unemployment, and one long-term follow-up study found that more than 80% of people diagnosed with schizophrenia have some ongoing social disability [3].

Long-term antipsychotic treatment has been the norm for people diagnosed with schizophrenia and other recurrent psychotic disorders since the introduction of these drugs in the 1960s [4]. Recent data from the United Kingdom indicate that 97.5% of mental health service patients diagnosed with schizophrenia are prescribed at least one antipsychotic [5]. The practice is based on research believed to have established that continuous antipsychotic treatment reduces the risk of relapse. Interpreting the evidence is not straightforward, though, and other data are beginning to emerge that suggest that long-term treatment may have an adverse impact on levels of social functioning [6,7]. Is it time, therefore, to review the practice of antipsychotic maintenance treatment and question whether it should continue to be the default treatment strategy in people diagnosed with schizophrenia or similar psychotic disorders?

## Evidence for Maintenance Therapy

Evidence for the benefits of long-term antipsychotic treatment consists of trials comparing antipsychotic maintenance with antipsychotic discontinuation. In other words, a group of patients already stabilised on antipsychotics are randomly allocated either to continue drug treatment, or to have it withdrawn and, in most cases, replaced by placebo. As a whole, these trials show that patients who have medication withdrawn are more likely to have increased symptoms, usually defined as relapse. However, several commentators have pointed to issues that complicate the interpretation of these “discontinuation” trials [8–10].

Firstly, the fact that antipsychotics, like other drugs, have withdrawal effects has not been adequately acknowledged in trial design or interpretation. Patients allocated to antipsychotic discontinuation are vulnerable to experiencing antipsychotic withdrawal symptoms, which commonly include anxiety and agitation, and which may be mistaken for a relapse of the underlying condition. This possibility is exacerbated by the fact that there are no agreed-on criteria for relapse. Many studies rely on clinical judgement and others use definitions involving small changes on rating scales scores. These all include non-specific items, such as agitation and hostility, likely to be exaggerated by the physiological changes accompanying antipsychotic withdrawal. Only a minority of studies specify changes in positive psychotic symptoms [11]. Although withdrawal symptoms would be expected to be less prolonged than a genuine relapse, in fact, we know little about their course. It is possible they might persist for long periods following long-term treatment, given evidence on other drugs [12].

Moreover, the experience of antipsychotic withdrawal may, in itself, make a relapse of the underlying condition more likely. The phenomenon of withdrawal-induced relapse has been shown convincingly in relation to lithium in people with bipolar disorder. Patients who stop long-term lithium treatment are more at risk of having a relapse than they were before they started it [13,14]. There is some evidence of a similar effect following antipsychotic withdrawal in people with schizophrenia. Relapses cluster around the point of withdrawal in most studies, for example, and one meta-analysis found that gradual withdrawal reduced the risk of relapse [15], although a recent meta-analysis did not replicate this finding [11]. However, it may be the case that withdrawal over an average of four weeks, as in the included studies in the most recent analysis, is not gradual enough for people who have been on medication for many years. Alternatively, withdrawal-related effects may occur however carefully treatment is withdrawn.

Finally, there is evidence that antipsychotic withdrawal may occasionally be “psychotogenic” in itself, including case reports where withdrawal of antipsychotic-like drugs precipitated psychotic episodes in people with no history of psychiatric disorder [16]. Studies of clozapine show that people can become more severely psychotic after discontinuing clozapine than when they started it [17,18]. The short half-life of clozapine is likely to be relevant to this effect, but some data suggest that a withdrawal-induced psychosis may occasionally occur with other antipsychotics [19].

Therefore, antipsychotic discontinuation studies may partially, or even wholly, reflect the adverse effects of antipsychotic withdrawal, rather than the benefits of initiating maintenance treatment. Further problems with existing studies include the fact that most focus on relapse as their principle outcome, with few providing data on other outcomes such as functioning, quality of life, work performance, or aggressive behaviour and violence. In a recent meta-analysis, for example, only three studies provided data on quality of life. Differences favoured maintenance treatment overall, but the longest study (which was negative) lasted only eight months. Only two studies reported data on employment, with no difference between maintenance and discontinuation groups, and no data on functioning was reported. Five studies reported on aggressive behaviour, which was rare, but more common in people who had antipsychotics discontinued overall. No study lasted longer than a year, however, and most involved abrupt discontinuation [11].

We also know little about how patients balance the risk of relapse against other outcomes. If relapse is not severe, for example, and side effects of drugs are experienced as disabling, patients may accept relapse as a price worth paying. Although some commentators have suggested that relapses worsen outcome [20], evidence from discontinuation trials indicates that symptoms return to normal when drug treatment is re-instated [21,22]. Suggestions that relapse is indicative of a neurotoxic process are also not substantiated by clinical or neurobiological evidence [23–25].

Another limitation of existing studies is that few provide long-term data to match the duration for which most patients are treated. This is a particular concern because of the association of relapse or deterioration with the point of discontinuation. Evidence from the few trials with follow-up lasting over a year suggests that the difference in relapse rates between patients maintained on antipsychotics and those who are discontinued lessens over time [11].

## Adverse Effects of Long-Term Antipsychotics

Long-term antipsychotic therapy is associated with common and potentially serious complications, so any uncertainty about the benefits of such treatment is a major concern. Tardive dyskinesia, a neurological condition involving involuntary movements associated with cognitive impairment [26], remains common. Recent studies find it affects approximately 4%–5% of people per year who take antipsychotics [27,28]. It is known to be irreversible in some cases and can occur after a few months of treatment [29,30]. Tardive dyskinesia has been recognised for many years, but recent animal and clinical studies have revealed that long-term antipsychotic treatment is associated with reduced brain weight and volume [31,32]. Although we are not certain whether these findings have functional implications, most studies suggest that brain volume reduction is associated with reduced cognitive performance [33].

Antipsychotics are cardio-toxic and associated with sudden cardiac death [34,35]. Some studies report an increased risk of all-cause mortality, even after controlling for other risk factors [35,36], but others report lower risks with long-term treatment [37]. Most antipsychotics cause weight gain of some degree, and some of the “atypical” antipsychotics can cause extreme weight gain, glucose and lipid abnormalities, and diabetes [38,39]. Metabolic abnormalities develop within days of drug initiation [40] and have occasionally been reported to be irreversible [41,42]. Antipsychotics are also reported to be unpleasant to take, causing emotional blunting and sexual dysfunction, among other undesired effects [43,44].

## New Evidence on Long-Term Treatment

Fifteen- and twenty-year outcomes from a long-term cohort study involving people with early psychosis have recently been published. The data suggest that people who take antipsychotics on a continuous basis have poorer outcomes than people who have periods of not taking antipsychotics [4]. The effect persisted after controlling for early prognostic factors [45]. Moreover, participants diagnosed with schizophrenia, who were not taking antipsychotics, showed better outcomes than those diagnosed with other forms of psychosis (usually associated with a better prognosis), who were on continuous treatment [27]. Nevertheless, confounding by severity is always relevant in a naturalistic study.

The results are supported, however, by data from a seven-year follow-up of an antipsychotic discontinuation study. This study, conducted in the Netherlands with people following resolution of a first episode of psychosis, represents the first really long-term follow-up of a randomised cohort. It consisted of a comparison between maintenance treatment and a flexible and gradual antipsychotic reduction and discontinuation strategy. Only 22% of participants stopped antipsychotics successfully during the randomised treatment period, and 46% never managed to stop them at all. At the 18-month follow up, relapses were more frequent in the group randomised to the discontinuation strategy, in line with other studies [46]. At seven-year follow-up, however, relapses had equalised between the groups, and participants originally randomised to antipsychotic reduction and discontinuation were twice as likely to show a full social recovery as those allocated to the maintenance group (40% versus 18%; *p* = 0.004) [6]. Symptomatic remission, however, which was achieved by 68% of the original sample, did not differ between the groups (*p* = 0.78). At follow-up, use of antipsychotics in the antipsychotic reduction and discontinuation group was lower, with 42% using no antipsychotics or very low doses (less than 1 mg of haloperidol equivalent per day) compared to 24% of the maintenance group. A post hoc analysis by treatment received over follow-up, regardless of randomised group, demonstrated antipsychotic discontinuation (or reduction to very low doses) was associated with higher rates of recovery (53% versus 17%; *p* < 0.001).

The lower neuropsychological functioning demonstrated in participants in the maintenance group at 18 months may indicate the mechanism of the effect on functional recovery detected at seven-year follow-up [47]. Other possible factors, including drug adherence and service engagement, should also be considered, however, although no such data have yet been presented. The Dutch study was conducted in young people who had had only one episode of psychosis. Only 50% of eligible patients agreed to participate. Results may not, therefore, be generalizable to people with longer-term conditions. A recent meta-analysis of antipsychotic maintenance trials found no difference in results between those conducted in people with a first episode and those with recurrent psychotic disorders, however [11].

## The Future

The majority of people who experience more than one episode of psychosis are currently recommended to remain on long-term antipsychotic treatment, with little guidance about whether the treatment should ever be stopped, and if so, how this should be done. Many patients find this approach unacceptable, and stop of their own accord without support, which likely leads to the complications of sudden medication withdrawal, including relapse.

The studies used to justify current clinical practice do not provide reliable data about the costs and benefits of long-term antipsychotic therapy. In particular, questions remain about how maintenance treatment affects people’s overall functioning over the long term, with some indications it may be detrimental for some people. There is abundant evidence that long-term antipsychotic treatment is associated with other serious and disabling adverse effects.

We need to do more research to establish the pros and cons of long-term antipsychotic treatment for people with one or more episodes of psychosis or schizophrenia. Further studies that evaluate a gradual and individualised approach to antipsychotic discontinuation are particularly important, both in people with first episode psychosis, and more challengingly, in people with recurrent conditions. Such studies need to include assessment of outcomes other than relapse and could assess what additional support might facilitate patients to successfully reduce their antipsychotic burden. Longer-term follow-up of five to ten years is required to reflect the duration of treatment in clinical practice. Research on treatments for other medical conditions demonstrates this can be achieved when it is prioritised [48,49].

Response to long-term antipsychotic treatment is likely to be heterogeneous, although so far there has been little success in identifying factors that might predict successful discontinuation [11]. Existing research rarely distinguishes people who recover and are symptom-free between episodes from those who have ongoing positive psychotic symptoms. In the former situation, long-term antipsychotic treatment is aimed solely at preventing relapse, whereas in the latter, long-term treatment may be a form of symptom control, instead of, or in addition to, its desired prophylactic effect. The considerations involved in these situations may be different, and research needs to identify the varying pros and cons of long-term treatment for the two groups. For example, in people who recover completely, the adverse consequences of having reduced social or neuropsychological function may be more significant than for those people who are already somewhat disabled by ongoing symptoms.

While we await the results of further long-term discontinuation studies, I suggest we need to reconsider antipsychotic maintenance treatment as the default strategy for people with recurrent psychotic disorders. In 1976, two leading psychiatrists felt that the cost-benefit ratio of long-term antipsychotic medication was often not favourable for patients and recommended that “every chronic schizophrenic outpatient maintained on antipsychotic medication should have the benefit of an adequate trial without drugs” [50]. Recent evidence suggests that, when risks allow, modern-day clinicians and patients could also consider this option.
